# An Ab Initio and Kinetic Monte Carlo Simulation Study of Lithium Ion Diffusion on Graphene

**DOI:** 10.3390/ma10070761

**Published:** 2017-07-06

**Authors:** Kehua Zhong, Yanmin Yang, Guigui Xu, Jian-Min Zhang, Zhigao Huang

**Affiliations:** 1 Fujian Provincial Key Laboratory of Quantum Manipulation and New Energy Materials, College of Physics and Energy, Fujian Normal University, Fuzhou 350117, China; yym@fjnu.edu.cn (Y.Y.); xuguigui82@126.com (G.X.); jmzhang@fjnu.edu.cn (J.-M.Z.); 2 Concord University College, Fujian Normal University, Fuzhou 350117, China; 3 Fujian Provincial Collaborative Innovation Center for Optoelectronic Semiconductors and Efficient Devices, Xiamen 361005, China

**Keywords:** graphene, diffusion coefficients of Li ion, first-principle calculations, Kinetic Monte Carlo

## Abstract

The Li^+^ diffusion coefficients in Li^+^-adsorbed graphene systems were determined by combining first-principle calculations based on density functional theory with Kinetic Monte Carlo simulations. The calculated results indicate that the interactions between Li ions have a very important influence on lithium diffusion. Based on energy barriers directly obtained from first-principle calculations for single-Li^+^ and two-Li^+^ adsorbed systems, a new equation predicting energy barriers with more than two Li ions was deduced. Furthermore, it is found that the temperature dependence of Li^+^ diffusion coefficients fits well to the Arrhenius equation, rather than meeting the equation from electrochemical impedance spectroscopy applied to estimate experimental diffusion coefficients. Moreover, the calculated results also reveal that Li^+^ concentration dependence of diffusion coefficients roughly fits to the equation from electrochemical impedance spectroscopy in a low concentration region; however, it seriously deviates from the equation in a high concentration region. So, the equation from electrochemical impedance spectroscopy technique could not be simply used to estimate the Li^+^ diffusion coefficient for all Li^+^-adsorbed graphene systems with various Li^+^ concentrations. Our work suggests that interactions between Li ions, and among Li ion and host atoms will influence the Li^+^ diffusion, which determines that the Li^+^ intercalation dependence of Li^+^ diffusion coefficient should be changed and complex.

## 1. Introduction

Despite the comprehensive commercial use of C-based materials as the negative electrode in Li-ion batteries for a long time, its electronic and ionic transport properties are not understood well. These fundamental transport properties are very important for designing high power and energy density Li-ion batteries. Graphene is one of the most important C-based negative electrode materials. It is a two-dimensional (2D) film containing carbon atoms with honeycomb lattice [1]. It has many interesting physical properties, such as quantum electronic transport [2], massless Dirac fermions [3] and quantum anomalous hall effect [4], and has potential applications in physics, chemistry, energy and so on. Therefore, it has attracted a lot of research interest around the world since it was found in 2004 [5]. One of its many important applications is the potential application on Li ion batteries and other electrochemical storage devices [6,7,8,9]. Experimental results have demonstrated that graphene nanosheets have a good cyclic performance, and its specific capacity can reach 460 mA h g^−1^ after 100 cycles [10]. Due to its high lithium storage capacity, good chemical stability, high conductivity, and good mechanical flexibility, graphene has been considered to be a suitable electrode alternative of battery [11,12].

Lithium-ion diffusion coefficient is a very important parameter in determining how fast a lithium ion battery can be cycled. Moreover, its determination will contribute to understanding the ionic transport mechanism in batteries. Several reports have showed that there are many techniques [13,14,15,16,17,18,19] to estimate the diffusion coefficient of ions. Among them, electrochemical impedance spectroscopy (EIS) [16,17] is considered as a very powerful technique that is often used in experiments. In EIS technique, the chemical diffusion coefficient is calculated based on the solution of the Warburg impedance response [20,21,22]. However, unfortunately, because there are many experimental factors affecting the Lithium-ion diffusion coefficient in a battery, along with the extremely complex analysis of experimental data, the reported experimental results for diffusion coefficients of lithium ions vary a lot. For example, for a variety of composite-graphite electrode architectures, lithium-ion diffusion coefficients have been reported to vary from 10^−6^ cm^2^ s^−1^ to 10^−16^ cm^2^ s^−1^ [23,24,25,26,27,28,29,30,31]. 

Theoretically, using the density functional theory (DFT) method, Tachikawa [32] studied the lithium ion diffusion on a fluorine terminated graphene surface. The result showed that at around room temperature the Li ion moved freely on the surface, but it did not move towards the edge of surface. Deya et al. investigated the role of nitrogen and boron doping on Li ion diffusion along the vertical direction of defective graphene by using first-principles calculations to evaluate the energy barrier [33]. They found that the atomic doping would reduce the energy barrier. Uthaisar et al. [34] investigated Li atoms diffusion on the graphene and graphene nanoribbons by means of density functional theory. They found that Li adatoms would diffuse toward the edges while Li diffusion channels appear along the ribbon axis and their energy barriers were smaller than those on graphene. Some researchers [35,36] have devoted their study to Li diffusion on pristine and defective graphene in both directions perpendicular and parallel to graphene using first-principles calculations. It is found that defects would bring down the energy barrier in the vertical direction of graphene nanosheets and consequently enhance the diffusion of Li. However, even though there have been many studies on Li diffusion in graphene, they basically focus solely on the diffusion behavior of a single particle without considering inter-particle interaction. In fact, the interaction between particles is always present, which must affect Li diffusion processes. Furthermore, due to the absence of dependable experimental techniques and theoretical methods, Li diffusion in graphene is still not understood well.

To effectively simulate the time evolution of a system, Kinetic Monte Carlo (KMC) simulation is designed. Taking the energy barriers from DFT calculations as input parameters, KMC simulation has been used to research atom desorption/adsorption processes on the surface [37], atom diffusion in bulk [38,39], and so on. In this work, we study the diffusion coefficient of lithium ion in Li^+^-adsorbed graphene systems by combining first-principle calculations with KMC simulations, and mainly investigate the impacts of Li^+^ concentration and temperature on the diffusion coefficients of Li^+^. Especially, the interactions between Li ions are considered. Once interactions between Li ions are taken into consideration, due to its sensitive dependence of near atom environment, the Li^+^ migration energy barrier might change at any time in Li^+^’s migration process. Thus, it is hard to get the energy barriers momentarily. However, Li^+^ migration energy barriers used as input parameters in KMC simulations must be obtained to assess the diffusion coefficient of a lithium ion. To estimate the energy barriers in Li^+^’s migration process well, based on the energy barriers obtained from first-principle calculations for single-Li^+^ and two-Li^+^ adsorbed systems, we deduce a new equation to evaluate the energy barriers for Li^+^-adsorbed systems with more than two Li ions. Moreover, according to the above-calculated energy barriers and KMC, the temperature and concentration dependences of diffusion coefficients of Li^+^ were studied. The calculated results mean that the temperature dependence of the acquired diffusion coefficients of Li^+^ were fitted to the Arrhenius equation, rather than meeting that from EIS technique, which had usually been used to estimate diffusion coefficients in experiments. At the same time, we also found that the Li^+^ concentration dependence of the calculated diffusion coefficients of Li^+^ also did not fully conform to that expected from the EIS technique.

## 2. Methods

In our work, the first-principle calculations were implemented by using Vienna ab initio simulation package (VASP), and the projector augmented wave method was considered in the electron-ion interactions [40,41,42,43,44,45,46]. The exchange-correlation energy was calculated based on Perdew-Burke-Ernzerhof (PBE) [47] formulation of generalized gradient approximation (GGA) [40]. The cut-off energy was chosen as 450 eV. Considering the magnetism of the Li ion, the spin polarization was included. By the calculations, the lattice constant for graphene was obtained to be 2.459 Å, which is near the experimental value of 2.46 Å. A 3 × 3 × 1 Monkhorst-Pack *k*-mesh was used for 7 × 7 × 1 supercell of graphene primitive cell. Moreover, a vacuum separation of 18 Å was employed to avert the artificial coupling role between neighboring layers. The geometry optimizations were implemented until the force acting on each atom was less than 0.02 eV/Å. To study Li ion kinetics on graphene, the total energies along the possible diffusion pathways were calculated, and the diffusion barriers of Li ion were estimated as the energy difference between the configurations in which the Li ion was placed at the initial position and the most unstable position on the diffusion pathway [35,48]. The diffusion barrier was applied to estimate the macroscopic Li^+^ diffusion coefficient on graphene using KMC simulations.

The detailed KMC algorithm follows the conventional procedure [49,50]. In the KMC simulation, the transition state theory (TST) is applied to describe the influences of interactions among neighboring Li ions and temperature. According to TST, an ion’s migration from an initial site to its adjacent vacancy is achieved by experiencing a transition state. There is an energy barrier *E*_m_ separating the two corresponding states before and after migration. The migration of the Li ions follows the general KMC procedures: (1) Determine all possible migration sites. With regard to the current atomic configuration, the first-nearest-neighbor vacant sites were considered as possible migration sites. (2) Identify a series of the transition rates (*p*_i_) for all possible migration states. Based on TST theory, the transition rate is defined as follows [51,52]:

(1) $$p_{i}=p\;\exp\left(-E_{m}/k_{B}T\right),$$

where, the preexponential factor $p=2k_{B}T/h$ is the jump frequency. *E*_m_ denotes the migration energy barrier which can be obtained from the first-principle calculations. *h*, $k_{B}$, and *T* are Planck’s constant, the Boltzmann constant and temperature (*K*), respectively. (3) Calculate a accumulation $P_{j}=\overset{j}{\underset{i=1}{\sum }}p_{i}$, with *j* = 1, …, *n* at the current position of a selected Li^+^, respectively. Here, *n* denotes the total number of the possible jumping sites. In fact, *j* does also mean *j*th jumping direction. As the neighboring site is occupied, a zero transition rate is set. Next, a uniform random number *u* from the interaval [0, 1] is drawn, and then the *j*th jumping direction was selected according to *P*_j-1_ < *u P*_n_ < *P*_j_. Thus, the migration event was generated, and the change of its neighbors caused by migration was then updated. Then the simulation moves into the next step. At each step of the algorithm, the simulation time *t* is incremented by Δ*t* which is given by

(2) $$\Delta t=-P_{n}^{-1}\ln u.$$

In KMC simulation, according to the Einstein relation the diffusion coefficient could be decided by: [51,52]

(3) $$D=\underset{t\to \infty }{\lim}\frac{<r{\left(t\right)}^{2}>}{4t},$$

where *t* is the simulation time and $<r{\left(t\right)}^{2}>$ is the mean squared displacement of Li. Each KMC simulation step contains *N*_Li_ steps (*N*_Li_ is the total number of Li ions), and the mean squared displacement is the average for all Li ions. The sum of all time increments for each jump is defined as the total time increment.

**Figure 1:**
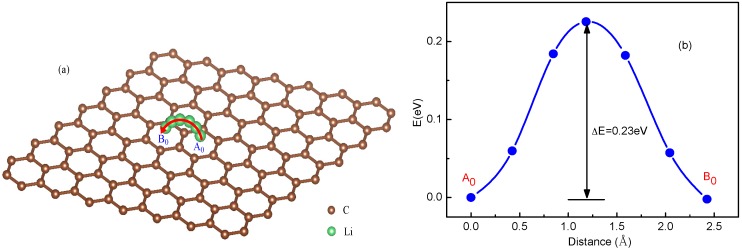
Schematic representation and total energy curve of a single Li ion diffusion on graphene following *A*_0_-*B*_0_ path.

Graphene is a single atomic layer of carbon, and its structure is shown in Figure 1. The graphene size with 14 × 14 Li^+^ occupied sites was used in the KMC simulations, using periodic boundary conditions for the simulation cell. Using the simulation size with 14 × 14 Li^+^ occupied sites, good calculated results can be obtained, because it has been tested that the simulated results from the sizes of 21 × 21 and 28 × 28 Li^+^ occupied sites are almost the same as those from the sizes of 14 × 14. The simulation temperature range is set to 230–330 K. For all simulations, 5000 MC steps are used, and the first 2000 MC steps are disregarded. Thus, the system is allowed to relax to the equilibrium state. After it reaches equilibrium, the average value of the last 3000 MC steps is the diffusion coefficient. For Li^+^-adsorbed graphene systems, Li^+^ concentration range considered to be from 0.01 to 0.33 monolayer (ML) in KMC simulations. The interactions between Li ions were considered in estimation of macroscopic diffusion coefficient of Li ions. Since the interaction between Li ions decreases with the increase of distance, in this paper, to simplify calculations, we only considered the nearest, second-nearest and third-neighbor interactions. For single-Li^+^ and two-Li^+^ adsorbed graphene systems (here, two-Li^+^ means that system contains one Li ion plus another Li ion), its migration energy barrier was directly obtained from total energy calculation of the systems using first-principle calculations. For Li ion with more than two Li neighbor ions, their migration energy barriers were derived from the results of the single-Li^+^ and two-Li^+^ adsorbed graphene systems according to an equation. The detailed introduction can be found below.

## 3. Results of DFT and KMC Calculations

### 3.1. Migration Energy Barrier in Single-Li^+^ and Two-Li^+^ Adsorbed Graphene Systems

Generally, there are three high symmetry sites considered for lithium ion adsorption on the graphene: top, hollow and bridge sites. The top site denotes the points up a carbon atom, and the hollow site denotes the center of a hexagon. Moreover, bridge site means the point up the midpoint of a carbon-carbon bond. The stability of a Li^+^-adsorbed graphene system is generally described by the following adsorption energy *E*_ad_: [35]

(4) $$E_{\mathrm{ad}}=\left(E_{\mathrm{ad}+\mathrm{Gr}}-E_{\mathrm{ad}}-E_{\mathrm{Gr}}\right),$$

where $E_{\mathrm{ad}+\mathrm{Gr}}$ denotes the total energy for Li^+^-adsorbed graphene system with a Li ion adsorbed on the graphene. $E_{\mathrm{ad}}$ and $E_{\mathrm{Gr}}$ are the energies of an isolated Li ion and the isolated graphene, respectively. The calculated adsorption energies (−3.147 eV, −2.902 eV and −2.922 eV for hollow, top and bridge sites, respectively) show that the most stable site locates the hollow one for the Li ion to be adsorbed on, which is consistent with previous research results [35]. Fan et al. found that for the single-layer graphene with the Li/C ratio being less than 1/6, Li was more likely to adsorb on the hollow site, and too high Li concentration would lead to more complex adsorption, such as no adsorption on the hollow site and a double adsorption [35]. Therefore, in this paper, only the Li^+^-adsorbed graphene systems with Li^+^/C ratio less than 1/6 were studied. Moreover, in the following study of Li^+^ diffusion on graphene by KMC simulations, Li ions migrated between hollow sites for all Li^+^-adsorbed graphene systems.

The diffusion energy barrier for Li ions can be obtained from the total energy curves of the Li^+^-adsorbed graphene systems along the diffusion pathway [35,48]. For a system with a single Li^+^, considering that Li^+^ is able to migrate via jumping between neighboring hollow sites, the total energy curve along *A*_0_-*B*_0_ diffusion pathway was shown in Figure 1. From the energy curve in Figure 1b, a Li ion diffuses by overcoming an energy barrier Δ*E* = 0.23 eV, which is near that of 0.311 eV in Ref. [35], corresponding to an energy maximum point locating in the middle of diffusion pathway. 

**Figure 2:**
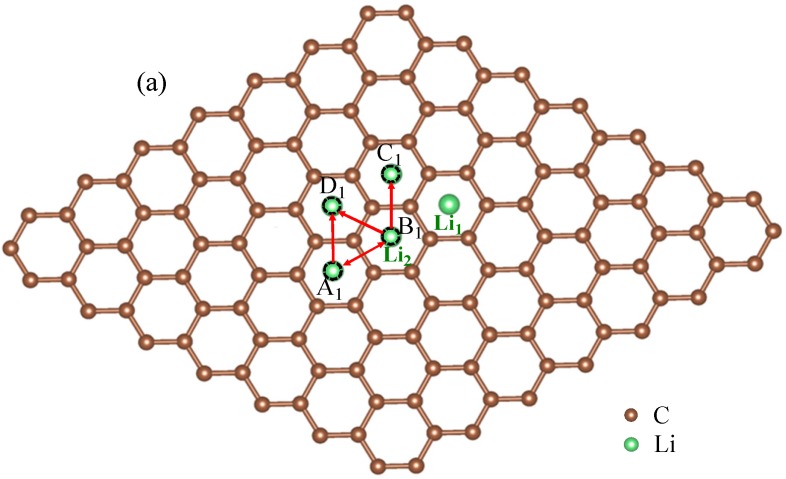

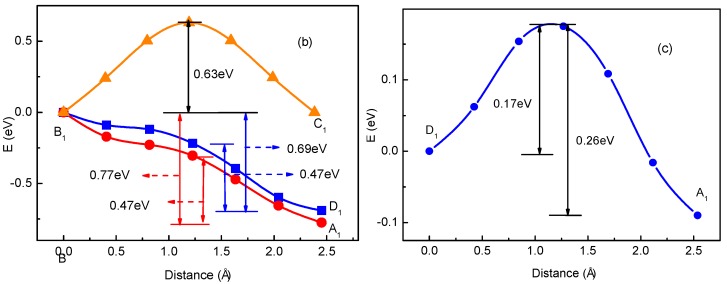
Schematic representations (**a**) and total energy curves for the system with two Li ions on graphene along (**b**) paths B_1_-C_1_, B_1_-A_1_ and B_1_-D_1_; (**c**) path D_1_-A_1_. Li^+^_1_ is migrating with Li^+^_2_ fixed.

For rare Li ions adsorbed on graphene, Li^+^ diffusion can be seen as the migration behavior for non-interacting particles. However, with the increase of Li^+^ concentration, the interaction between Li ions will in fact appear inevitably in the experiment. Then, considering interaction between Li ions becomes important. To study the interaction between Li ions, we calculated total energies for the two-Li^+^ adsorbed graphene systems that Li_1_ ion is fixed while Li_2_ ion is migrating, as shown in Figure 2. To simplify, we just considered the interactions between the nearest, second-nearest and third-neighbor Li ions. In Figure 2a, there are three possible migration pathways (labeled as B_1_→A_1_, B_1_→D_1_, B_1_→C_1_) for Li_2_ ion in its migration behavior, when another Li_1_ ion has already existed on Li^+^_2_’s nearest-neighbor site. A_1_, D_1_ and C_1_ sites correspond to the nearest, second-nearest and third-neighbor sites of Li_1_ ion, respectively. According to the total energy curve shown in Figure 2b, it is clearly found that D_1_ and C_1_ sites are more energetically favorable than A_1_ site in Li^+^_2_’s migration behavior. Li^+^_2_ jumping from B_1_ to C_1_ site along migration pathway B_1_→C_1_ needs to get over an energy barrier of about 0.63 eV. However, for the other two migration pathways B_1_→D_1_ and B_1_→A_1_, Li^+^_2_ can spontaneously jump from B_1_ to D_1_ (A_1_) sites without overcoming the energy barrier. However, for Li^+^_2_’s reverse migration processes from D_1_, C_1_ and A_1_ sites to B_1_ site, no matter which of the three pathways, all Li^+^_2_ needs to get over the high barriers of 0.63, 0.69 and 0.77 eV for paths C_1_→B_1_, D_1_→B_1_ and A_1_→B_1_, respectively. Figure 2c shows the total energy curve for Li^+^_2_ jumping between the second-nearest and third-neighbor sites of Li^+^_1_ (D_1_ and A_1_). Li^+^_2_ needs to get over an energy barrier about 0.17 eV when it jumps from D_1_ to A_1_. However, to jump from A_1_ to D_1_, it will get over a larger barrier of 0.26 eV, which is larger than that for a single Li^+^ diffusion on graphene (0.23 eV). Compared with the migration behavior for a single Li^+^’s diffusion on graphene, the migration behavior for the system with two Li ions becomes more complex. Obviously, these complex migration behaviors are as a result of the influences of Li^+^_1_ located nearby. Furthermore, the differences among migration behaviors for Li^+^_2_ along paths B_1_↔D_1_, B_1_↔C_1_, B_1_↔A_1_ and A_1_↔D_1_, as shown in Figure 2b,c, further prove that interaction between close Li ions will greatly influence the migration direction and energy barrier. Moreover, it indicates that the interaction between Li ions should be considerable, especially for the system with high Li^+^ concentration. The calculated results are similar to those in Refs. [51,52,53,54,55,56]. Takachi et al. found that Li-Li or Na–Na interaction resulted in the changing of the Li (Na)–graphene interaction, which gave rise to the enhancing energy barrier. Moreover, it was concluded that ideal single layer graphene is unsuitable to be used as an anode for Li-ion batteries [53].

For a multi-Li^+^ adsorbed graphene system with high Li^+^ concentration, Li ions would interact with each other through Coulomb repulsion. Such interactions of mutual Coulomb repulsion among many Li ions may play an important role on Li^+^’s migration energy barriers, consequently affecting their diffusion process on graphene. However, once the interactions among many ions are taken into account, the problem becomes very complicated. It is difficult to describe the interaction between Li ions on the energy barrier using first-principle calculations, because of Li^+^’s random nature of local bonding configurations. Li^+^’s arrangement changes at any time in diffusion process, so the corresponding Li^+^ migration barriers subsequently vary. To provide a fundamental understanding of the influence of Li^+^’s concentration on diffusion coefficient, we developed an equation to describe Li^+^ migration energy barriers for multi-Li^+^ adsorbed graphene system having Li^+^’s interactions in the following sections. It is well known that Coulomb interaction energy between two ions is inversely proportional to their distance. As the distance increases enough, the Coulomb interaction strength diminishes step by step. To simplify, here, we only consider the interactions between the nearest, second-nearest and third-neighbor Li ions to study the effects of Li^+^ concentration on Li^+^ diffusion coefficient for Li^+^-adsorbed graphene systems. 

### 3.2. Method for Energy Barrier Estimation 

**Figure 3:**
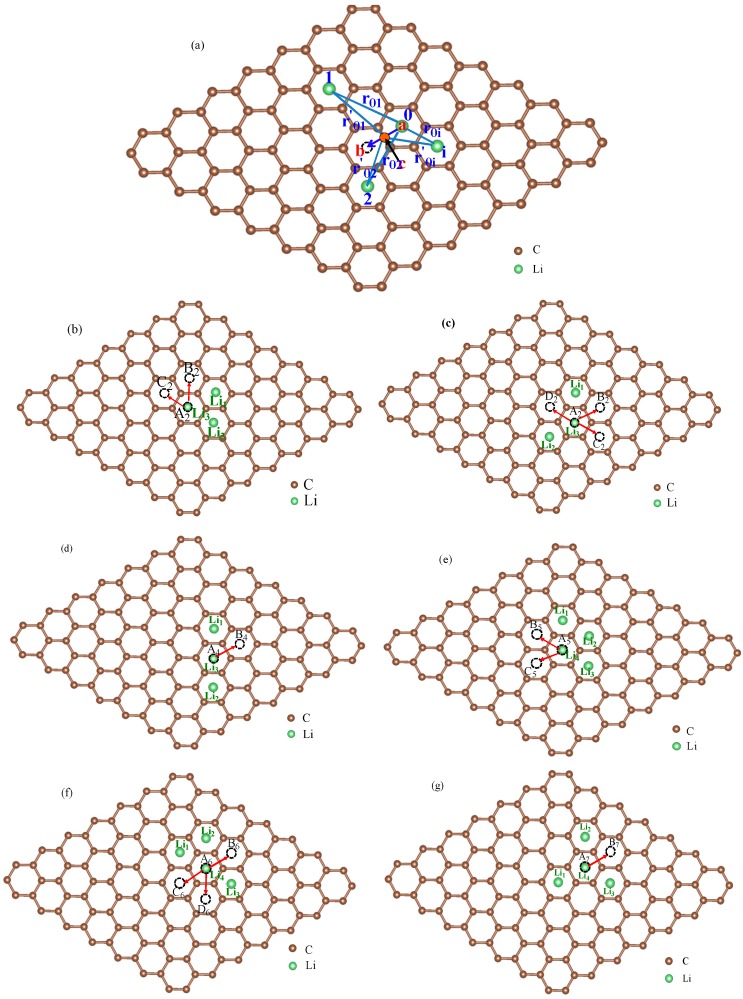
The schematic view of multi-Li^+^ adsorbed graphene system (**a**) and schematic representations for various possible Li^+^ arrangements and Li^+^’s possible migration pathways for three-Li^+^ and four-Li^+^ adsorbed systems with one Li^+^ migrating on graphene and the others fixed (**b**–**g**).

For multi-Li^+^ adsorbed graphene system, as shown in Figure 3a, the energy for transition states on migration path can be estimated as

(5) $$E_{a}=K_{1}\left(\frac{1}{r_{0,1}}+\frac{1}{r_{0,2}}+\frac{1}{r_{0,3}}+\ldots +\frac{1}{r_{0,i}}\right)+K_{2}E_{0,\mathrm{gr}}+H_{\mathrm{background}},$$

(6) $$E_{c}=K_{1}\left(\frac{1}{r_{0,1}^{\prime }}+\frac{1}{r_{0,2}^{\prime }}+\frac{1}{r_{0,3}^{\prime }}+\ldots +\frac{1}{r_{0,i}^{\prime }}\right)+K_{2}E_{0,\mathrm{gr}}^{\prime }+H_{\mathrm{background}}.$$

where $E_{a}$ and $E_{c}$ denote the total energies for multi-Li^+^ adsorbed graphene system with migrated Li ion (labeled as the *0*-th Li^+^) located on *a* and *c* sites of migration path, respectively. $E_{0,\mathrm{gr}}$ and $E_{0,\mathrm{gr}}^{\prime }$ are the energies for the systems which are only composed of graphene and the *0*-th Li^+^ located on *a* and *c* sites, respectively. $H_{\mathrm{background}}$ denotes the total energy for multi-Li^+^ adsorbed graphene system in which the *0*-th Li ion is removed. $K_{1}$ and $K_{2}$ are proportionality coefficients. $r_{0,i}$ and $r_{0,i}^{\prime }$ are the distances between the *0*-th and *i*-th Li ion. 

Combining Equation (5) with Equation (6), the energy difference $\Delta E_{\mathrm{ac}}$ for two transition states on the migration path can be evaluated as

(7) $$\Delta E_{\mathrm{ac}}=E_{a}-E_{c}=K_{1}\left[\left(\frac{1}{r_{0,1}}-\frac{1}{r_{0,1}^{\prime }}\right)+\left(\frac{1}{r_{0,2}}-\frac{1}{r_{0,2}^{\prime }}\right)+\ldots +\left(\frac{1}{r_{0,i}}-\frac{1}{r_{0,i}^{\prime }}\right)\right]+K_{2}\left(E_{0,\mathrm{gr}}-E_{0,\mathrm{gr}}^{\prime }\right).$$

For a two-Li^+^ adsorbed graphene system, such as the system in which only two Li ions (*0*-th and *1*-th Li ions) are adsorbed on the graphene, the energy for transition states on migration path can be estimated as

(8) $$E_{0,1}=K_{1}\frac{1}{r_{0,1}}+K_{2}E_{0,\mathrm{gr}}+E_{\mathrm{background}},$$

(9) $$E_{0,1}^{\prime }=K_{1}\frac{1}{r_{0,1}^{\prime }}+K_{2}E_{0,\mathrm{gr}}^{\prime }+E_{\mathrm{background}}.$$

where $E_{0,1}$ and $E_{0,1}^{\prime }$ are the energies of two-Li^+^ adsorbed graphene system with the *0*-th Li ion located on *a* and *c* sites on migration path, respectively. $E_{\mathrm{background}}$ is the energy of the two-Li^+^ adsorbed graphene system in which the *0*-th Li ion is removed. 

Combining Equation (8) with Equation (9), the energy difference $\Delta E_{0,1}$ for two transition states on the migration path can be evaluated as

(10) $$\Delta E_{0,1}=E_{0,1}-E_{0,1}^{\prime }=K_{1}\left(\frac{1}{r_{0,1}}-\frac{1}{r_{0,1}^{\prime }}\right)+K_{2}\left(E_{0,\mathrm{gr}}-E_{0,\mathrm{gr}}^{\prime }\right).$$

Analogously, for the two-Li^+^ adsorbed graphene systems which are only composed of the *2*-nd or *3*-rd or *i*-th Li ion and the *0*-th Li ion and graphene, the energy differences $\Delta E_{0,2}$ … and $\Delta E_{0,i}$ for the *0*-th Li ion jumping from *a* site into *c* site are written as:

(11) $$\Delta E_{0,2}=E_{0,2}-E_{0,2}^{\prime }=K_{1}\left(\frac{1}{r_{0,2}}-\frac{1}{r_{0,2}^{\prime }}\right)+K_{2}\left(E_{0,\mathrm{gr}}-E_{0,\mathrm{gr}}^{\prime }\right),$$

…

(12) $$\Delta E_{0,i}=E_{0,i}-E_{0,i}^{\prime }=K_{1}\left(\frac{1}{r_{0,i}}-\frac{1}{r_{0,i}^{\prime }}\right)+K_{2}\left(E_{0,\mathrm{gr}}-E_{0,\mathrm{gr}}^{\prime }\right).$$

Thus, combining Equation (7) with Equations (10)–(12), the energy difference for a multi-Li^+^ adsorbed graphene system can be derived as

(13) $$\Delta E_{\mathrm{ac}}=(\Delta E_{0,1}+\Delta E_{0,2}+\ldots +\Delta E_{0,i})-\left(i-1\right)\times \Delta E_{0,\mathrm{gr}}.$$

where, $\Delta E_{0,\mathrm{gr}}=K_{2}\left(E_{0,\mathrm{gr}}-E_{0,\mathrm{gr}}^{\prime }\right)$ is the energy difference for one-Li^+^ adsorbed graphene system with single Li^+^ migrating from *a* site into *c* site. Moreover, it was estimated to be about 0.23 eV, as shown in Figure 1. In Equation (13), the first term is the energy differences corresponding to two transition states on migration path for those two-Li^+^ adsorbed graphene systems. The second term is only related to the single-Li^+^ adsorbed graphene system with no interaction between Li ions. If two transition states correspond respectively to the activated state and initial state of the ionic hoping, the energy difference is the diffusion barriers. Thus, we can simply estimate the diffusion barriers for multi-Li^+^ adsorbed graphene systems from diffusion barriers of two-Li^+^ and single-Li^+^ adsorbed graphene systems shown in Figure 2b and Figure 1b according to Equation (13). Similarly, as the distance between two ions increases, the Coulomb interaction energy between them decreases. This can be also proved from Figure 1b, Figure 2b,c. When Li^+^_2_ jumps between the second-nearest and third-neighbor sites of Li^+^_1_ (D_1_ and A_1_), the energy barriers are about 0.17 and 0.26 eV, which is close to that for a single Li^+^ diffusion on graphene (0.23 eV). Compared with that for B_1_↔D_1_, B_1_↔C_1_ and B_1_↔A_1_ paths, the interaction between Li^+^_1_ and Li^+^_2_ evidently weakens a lot. Therefore, to simplify, it is reasonable to only consider the interactions between the nearest, second-nearest and third-neighbor Li ions in the study of the diffusion barriers for multi-Li^+^ adsorbed graphene systems. 

**Figure 4:**
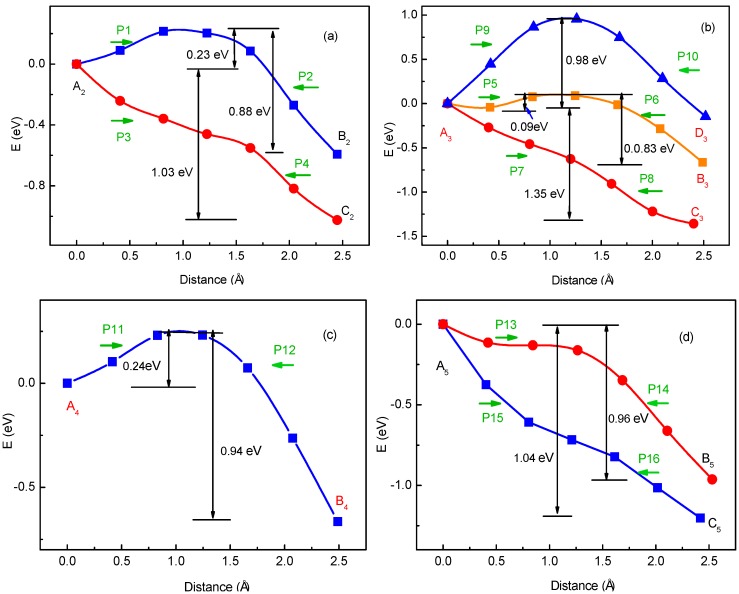

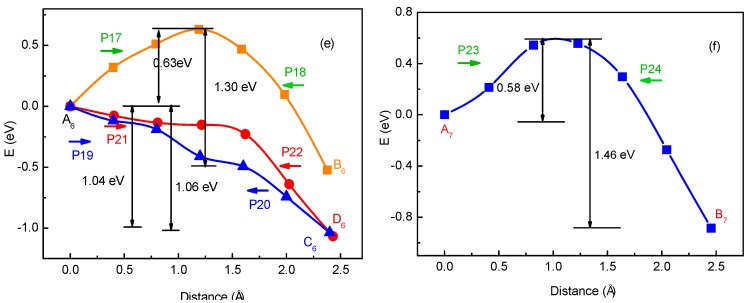
Total energy curves along Li^+^’s migration pathways labeled as P1–P24 for three-Li^+^ and four-Li^+^ adsorbed systems with Li^+^ arrangements shown in Figure 3b–g.

**Figure 5:**
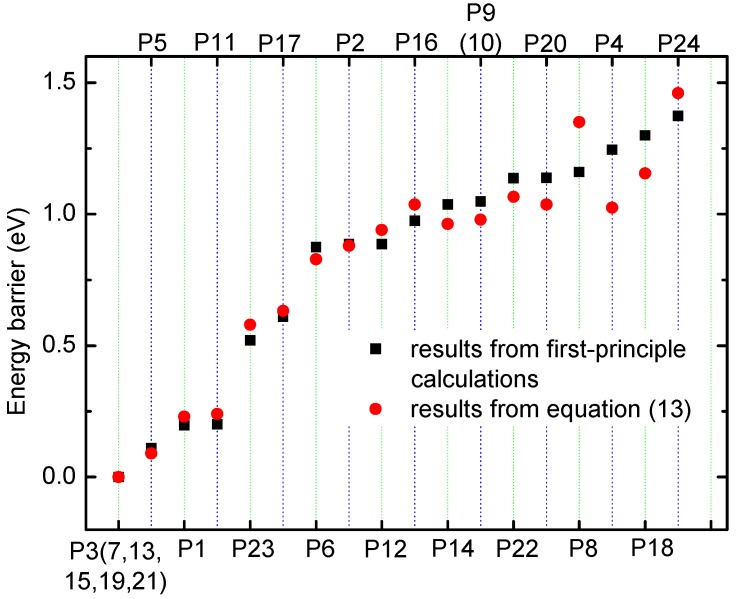
The energy barriers obtained from first-principle calculations and from Equation (13) for various Li^+^’s migration paths P1–P24 shown in Figure 4a–f.

To verify the method for energy barrier estimation, we presented that energy barriers can be estimated by combining Equation (13) and total energy curves obtained from first-principle calculations along various possible migration pathways for three-Li^+^ and four-Li^+^ adsorbed graphene systems. These systems have various possible Li^+^ arrangements. Because the Li^+^-adsorbed graphene systems considered in this paper only involve those with low Li^+^ concentration, as mentioned previously, Li^+^ basically has no more than three neighbors. This can also be confirmed in later sections. Therefore, total energy calculations are only performed on three-Li^+^ and four-Li^+^ adsorbed graphene systems. Figure 3b–g shows the systems with various possible Li^+^ arrangements and Li^+^’s various possible migration pathways, respectively. The migration pathways are labeled as P1–P24 in Figure 4a–f. For example, the migration pathway labeled as P1 denotes that Li^+^ migrates from A_2_ site to B_2_ site shown in Figure 3b, and P2 denotes Li^+^ migrates from B_2_ site to A_2_ site. Figure 4a–f shows the total energy curves along migration pathways corresponding to Figure 3b–g. Directly from the total energy curves, Li^+^ diffusion barriers are easily obtained, which are displayed in Figure 4a–f. From Figure 4a–f, it is indicated once again that the interaction between ions affects Li^+^’s migtation behavior greatly. As a result, the migrated Li ion energetically prefers to jump into the site far away from the other Li ions. Through another method, the diffusion energy barriers corresponding to Figure 3b–g can be also calculated by Equation (13) based on the total energy curves of single-Li^+^ and two-Li^+^ adsorbed graphene systems obtained from first-principles calculations, as found in Figure 1b and Figure 2b. Comparison of all diffusion barriers obtained by the two above methods for various Li^+^’s migration paths P1–P24 is shown in Figure 5. From the figure, it can be observed that the calculated results from both methods are consistent. The mean deviation Δ can be calculated as follows:

(14) $$\Delta =\frac{1}{N}\sum_{i=1}^{N}\frac{\left|D_{i,\mathrm{ab}}-D_{i,\mathrm{eq}}\right|}{D_{i,\mathrm{eq}}}\times 100\text{\%}.$$

*N* denotes the number of the samples. $D_{i,\mathrm{ab}}$ and $D_{i,\mathrm{eq}}$ result from first-principles calculations and Equation (13), respectively. The mean deviation *Δ* is calculated to be 9.97%. Therefore, the method that combining Equation (13) with the total energy curves of single-Li^+^ and two-Li^+^ adsorbed graphene systems from first-principles calculations, works well in estimating the diffusion barriers for multi-Li^+^ adsorbed graphene systems having Li^+^’s interaction. Using the diffusion barriers obtained from Equation (13), we can apply KMC to predict the macroscopic Li^+^ diffusion coefficient. 

Our calculated Li^+^ diffusion coefficients for the systems with rare Li^+^ adsorption (such as the system with Li^+^/C rate of 0.045) at room temperature are the value *D*_Li_ ~ 10^−7^ cm^2^⋅s^−1^. It agrees well with the result of previous theoretical and experimental research of intra-layer Li^+^ diffusion in graphite [57]. Moreover, it confirms that our KMC simulations are reliable. 

### 3.3. Concentration Effects on Li^+^ Diffusion Coefficient

The diffusion coefficient is an important parameter to determine the diffusion velocity of Li^+^, because it presents a more quantitative description of the diffusion features. In the experimental studies of electrochemical impedance spectroscopy (EIS), from the data analysis in the low-frequency region, the Li^+^ diffusion coefficient was estimated according to the following Equations [20,21,22]:

(15) $$D_{\mathrm{Li}}=\frac{R^{2}T^{2}}{2A^{2}n^{4}F^{4}\sigma^{2}C_{\mathrm{Li}}^{2}}$$

and

(16) $$Z_{\omega }=R_{\mathrm{ct}}+R_{1}+\sigma \omega^{-1/2}.$$

where, *D*_Li_ is Li^+^ diffusion coefficient, *C*_Li_ is the concentration of Li ion, *T* denotes the absolute temperature, *R* is the gas constant, *A* means the surface area, *n* denotes the number of electrons per molecule during oxidization, σ is the Warburg factor, and *F* is the Faraday’s constant, $Z_{\omega }$ is the Warburg impedance, and ω is frequency.

For the same temperature, we introduce a new parameter $Q_{1}$,

(17) $$Q_{1}=\frac{R^{2}T^{2}}{2A^{2}n^{4}F^{4}\sigma^{2}},$$

then Equation (15) can be rewritten as follows:

(18) $$D_{\mathrm{Li}}=Q_{1}C_{\mathrm{Li}}^{-2}.$$

The logarithm on both sides of the Equation (18) can be changed into:

(19) $$\ln\left(D_{\mathrm{Li}}\right)=\ln Q_{1}-2\ln(C_{\mathrm{Li}}).$$

To study the concentration effects on Li^+^ diffusion coefficient, the KMC calculated method was used to calculate Li^+^ diffusion coefficients for Li^+^-adsorbed graphene systems. Based on the previous research results [35] that too high Li^+^ concentration would lead to more complex adsorption, our research systems were only restricted to those Li^+^-adsorbed graphene systems with Li^+^/C ratio of less than 1/6 (equivalent to that *C*_Li_ is less than 0.337 ML). 

**Figure 6:**
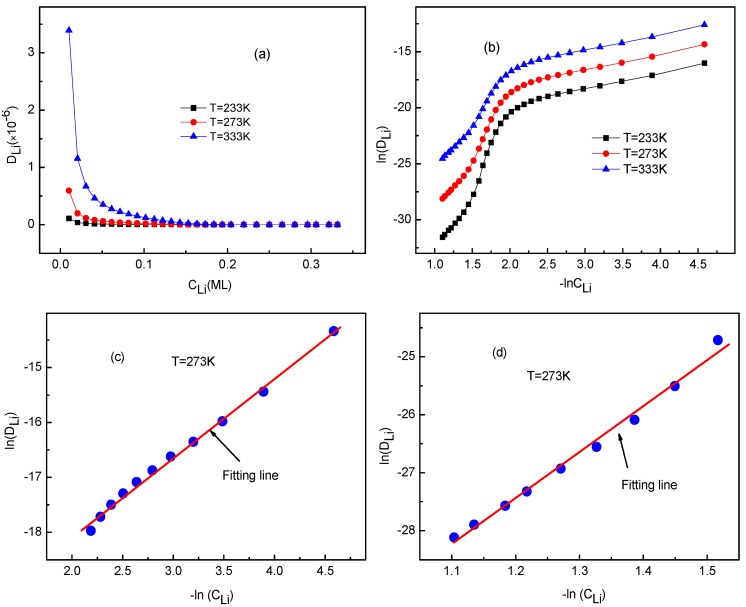
(**a**) The diffusion coefficient *D*_Li_ as a function of Li^+^ concentration *C*_Li_; (**b**) the logarithm of *D*_Li_ as a function of the logarithm of *C*_Li_, at *T* = 233, 273 and 333 K. The logarithm ln(*D*_Li_) as a function of −ln(*C*_Li_) for *C*_Li_ for (**c**) low concentration region, and (**d**) high concentration region at *T* = 273 K. The solid lines are the fitting ones.

The relationship between the calculated diffusion coefficient *D*_Li_ and Li^+^ concentration C_Li_ is shown in Figure 6a. From Figure 6a, it can be evidently seen that *D*_Li_ decreases quickly with increasing Li^+^ concentration in very low concentration region for all temperatures, and decreases slowly in comparatively high Li concentration regions. Figure 6b shows ln(*D*_Li_) as a function of −ln(*C*_Li_) at *T* = 233, 273 and 333 K. Unfortunately, it is quite clear that the calculated results do not fully meet the expected results from Equation (19), which has usually been used to estimate diffusion coefficients in an experiment. To get a deep insight into the relationship between the diffusion coefficient and concentration, the curve of ln(*D*_Li_) as a function of −ln(*C*_Li_) was divided into two parts: low concentration region (*C*_Li_ in range of 0.01–0.11 ML) shown in Figure 6c and relatively high concentration region (*C*_Li_ in range of 0.21–0.33 ML) shown in Figure 6d. In Figure 6c,d, the solid lines are the corresponding fitting lines. The fitting line slopes denote that the indexes β of *C*_Li_ are about equal to 1.44 and 7.95 for low and relatively high concentration regions at *T* = 273 K, respectively. Although the cases for other temperatures are not listed, the change trend for all other temperatures is the same. The slope β and correlation coefficients *r*_1_ for the linear fitting line for all temperatures are listed in Table 1. As seen in Table 1, the values of β in low concentration region (0.01–0.11 ML) are almost the same as that in temperature region of 233–333 K, which generally conforms to the law described by Equation (19). This indicates that in a low concentration region the relationship between *D*_Li_ and *C*_Li_ is roughly fitted to that of Equation (19). However, for a high concentration region (*C*_Li_ in range of 0.21–0.33 ML), the slope β gradually decreases from 8.94 at *T* = 233 K to 6.88 at *T* = 333 K. The value of β seriously deviates from 2. Therefore, the calculated results imply that Equation (15) could not be simply used to estimate the Li^+^ diffusion coefficient for all Li^+^-adsorbed graphene systems with various Li^+^ concentration, and it is only suitable for those systems with very low Li^+^ concentration in which the interaction between Li ions is very small. The calculated results are consistent with the experimental facts [23,53,58,59,60,61]. Takachi et al. studied the concentration dependence of Li^+^/Na^+^ diffusion in manganese hexacyanoferrates [53]. It is found that the potential barrier and diffusion coefficient of Na^+^ increases with increasing Na^+^ content, while those of Li^+^ decrease with increasing Li^+^ content, which is because the number of Li^+^ sites is three times that of the Na^+^ sites. In the low Li^+^ content, Li^+^ diffusion appears in Li^+^ diffusion path with a lower single-ion potential barrier. The experimental results indicate that the interaction between Li^+^/Na^+^ will play important roles in the diffusion coefficient. Takami and Piao et al. measured the diffusion coefficients of carbon electrodes [23,58]. They found that the diffusion coefficient decreased with increasing Li^+^ content. However, the changing slope of the diffusion coefficient is different for low and high intercalation Li^+^ regions. Similar changing relations were also found in Li_1-x_Ni_0.33_Mn_0.33_Co_0.33_O_2_ and Li_1-x_Ni_0.50_Mn_0.20_Co_0.30_O_2_ [59,60]. Chou et al. studied Li diffusion behavior in a-Li_x_Si and a-Li_x_Ge [61]. They found different Li^+^ content dependence of the Li^+^ diffusion coefficient. The Li^+^ diffusion coefficient increases with increasing Li^+^ content for a-Li_x_Si, and decreases with increasing Li^+^ content for a-Li_x_Ge. Therefore, interactions between Li ions, and among Li ion and host atoms will all influence the Li^+^ diffusion, which determines that the Li^+^ intercalation content dependence of Li^+^ diffusion coefficient should be changed and complex.

**Figure 7:**
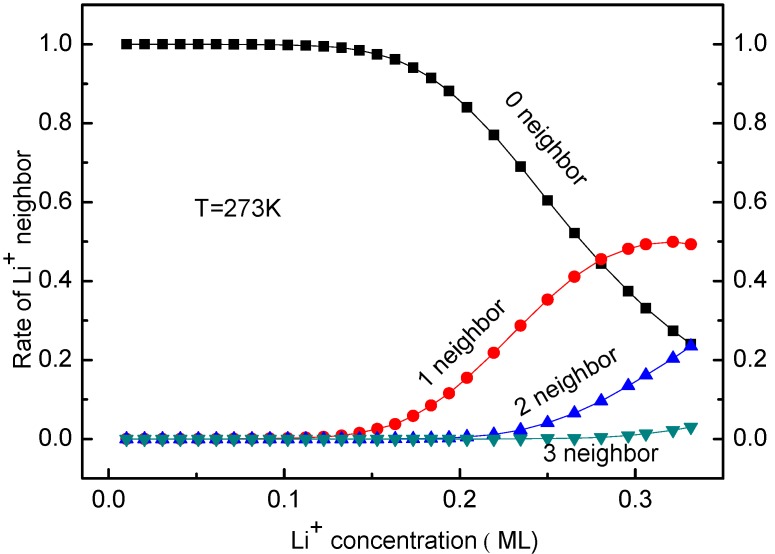
Rates of Li^+^ with 0–3 neighbors at *T* = 273 K for different Li ion concentration.

The occupying rates of Li ion having 0, 1, 2 or 3 neighbors can be calculated as well. The KMC simulated results indicate that these rates are unaffected by temperature. Moreover, the rates of Li having 4 or more neighbors are always zero for Li ion concentration < 0.33 ML. This coincides with the Li arrangements shown in Figure 3b–g, which was considered in the deriving process of Equation (13). Figure 7 shows the occupying rates of Li^+^ with 0–3 neighbors for all systems at *T* = 273 K for different Li ion concentration. In fact, the distribution of Li ion is decentralized in the system with low Li^+^ concentration, so it has no neighbors in a certain range. This corresponds to the case shown in Figure 7. With the increase of Li ion concentration, more Li ions are located close to each other. As a result, some Li ions have neighbors and the occupying rates with 1–3 neighbors increase, as shown in Figure 7. The temperature effects on the Li^+^ diffusion coefficient will be discussed in the next section. 

### 3.4. Temperature Effects on Li^+^ Diffusion Coefficient

Generally, according to the Arrhenius law the diffusion coefficient as a function of temperature is estimated as: [62,63]

(20) $$D_{\mathrm{Li}}\left(T\right)=D_{0}\exp\left(-\frac{E_{A}}{k_{B}T}\right).$$

$D_{0}$ is the pre-exponential factor, $E_{A}$ is activation energy. It should be noted that the activation energy in the Arrhenius equation reflects macroscopic activation energy and differs from the energy barrier for TST formulas. Take the logarithm on both sides of the Equation (20), it can be changed into:

(21) $$\ln\left(D_{\mathrm{Li}}\right)=-\frac{E_{A}}{k_{B}T}+\ln\left(D_{0}\right).$$

**Figure 8:**
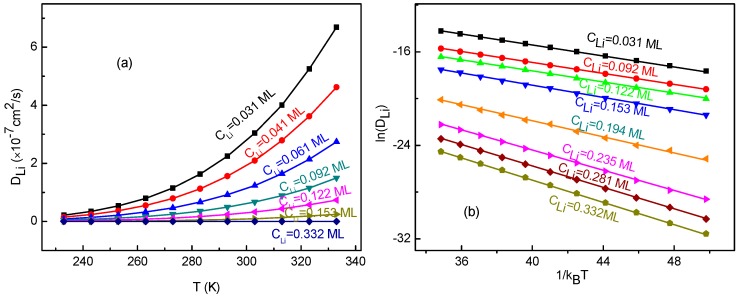
(**a**) The diffusion coefficient *D*_Li_ as a function of temperature *T*; (**b**) the logarithm of *D*_Li_ as a function of $1/\left(k_{B}T\right)$, and their fitting lines, for various Li^+^ concentration *C*_Li_.

In order to study the temperature effects on the Li^+^ diffusion coefficient, we have calculated the diffusion coefficients for the Li^+^-adsorbed graphene systems in a temperature range of 233–333 K. The calculated Li^+^ diffusion coefficient $D_{\mathrm{Li}}$ as a function of temperature is shown in Figure 8a. From the figure, it can be seen that $D_{\mathrm{Li}}$ increases with enhancing temperature for all Li^+^ concentrations. Moreover, with increasing Li^+^ concentration, the change of $D_{\mathrm{Li}}$ slows down quickly. Obviously, $D_{\mathrm{Li}}$ does not follow a quadratic relation shown in Equation (15). Additionally, the $\frac{1}{k_{B}T}$ dependence of $\ln\left(D_{\mathrm{Li}}\right)$, and their fitting curves are presented in Figure 8b. It can obviously be observed that there is a linear relationship between $\ln\left(D_{\mathrm{Li}}\right)$ and $\frac{1}{k_{B}T}$. This indicates that the obtained diffusion coefficients follow the Arrhenius equation, which is similar to other research results [62,63] rather than meeting that from Equation (15). The slope α corresponds to the negative of the macroscopic migration energy barrier $\left(-E_{A}\right)$. Furthermore, the slope α and correlation coefficient *r*_2_ for the fitting curves are listed in Table 2. The negative of the slope value of 0.23 eV for low Li^+^ concentrations agrees well with the migration energy barrier (0.23 eV) for a single-Li^+^ adsorbed graphene system. This meets the expectation because Li^+^ arrangements are divergent for the systems with low Li^+^ concentration. As a result, there is almost no interaction between ions. Thus, Li^+^ migrates like that for a single-Li^+^ adsorbed system. Therefore, the macroscopic migration energy barrier obtained from Arrhenius equation would very close to the value of a single-Li^+^ adsorbed system. With increasing Li^+^ concentration, the macroscopic migration energy barrier which depends on surrounding environment does increase, which leads to Li^+^’s diffusion becoming more difficult. It also confirms that the KMC simulation and the method for energy barrier estimation are reasonable and reliable.

## 4. Conclusions

In summary, combining first-principle calculations with KMC simulations, we determined the diffusion coefficient of lithium ions in Li^+^-adsorbed graphene systems. The impacts of Li^+^ concentration and temperature on the Li^+^ diffusion coefficients were mainly investigated. In the determination of the Li^+^ diffusion coefficient, the interactions between Li ions were taken into account. In the prediction of energy barriers, we deduce an equation based on those energy barriers obtained from first-principle calculations for single-Li^+^ and two-Li^+^ adsorbed systems to evaluate the energy barriers for Li^+^-adsorbed systems with more than two Li ions. The calculated results show that the temperature dependence of diffusion coefficients of Li^+^ fits well to the Arrhenius equation, rather than meeting the equation from the EIS technique, which has usually been used to estimate diffusion coefficients in an experiment. Moreover, our results also show that the Li^+^ concentration dependence of diffusion coefficients is roughly fitted to Equation (15) from the EIS technique in a low concentration region. However, it seriously deviates from Equation (15) in a high concentration region. Our work suggests that Equation (15) could not be simply used to estimate Li^+^ diffusion coefficient for all Li^+^-adsorbed graphene systems with various Li^+^ concentration. Moreover, these results have an important guiding significance in the experimental determination of the Li^+^ diffusion coefficient.

## Figures and Tables

**Table 1:** The slope β and correlation coefficient r_1_ for the linear fitting line between ln(*D*_Li_) and ln(*C*_Li_) in different region of concentration *C*_Li_ (ML) at various temperatures.

Concentration C_Li_ (ML)	Temperature T (K)						
		T = 233	T = 243	T = 253	T = 263	T = 273	T = 283
**0.01–0.11**	β	1.46 ± 0.04	1.46 ± 0.0	1.45 ± 0.04	1.45 ± 0.03	1.44 ± 0.03	1.44 ± 0.03
	*r*_1_	0.9936	0.9947	0.9942	0.9953	0.9949	0.9953
**0.21–0.33**	β	8.94 ± 0.38	8.69 ± 0.35	8.34 ± 0.34	8.04 ± 0.30	7.95 ± 0.28	7.80 ± 0.25
	*r*_1_	0.9859	0.9871	0.9866	0.9892	0.9899	0.9916
		**T = 293**	**T = 303**	**T = 313**	**T = 323**	**T = 333**	
**0.01–0.11**	β	1.43 ± 0.03	1.43 ± 0.03	1.44 ± 0.03	1.43 ± 0.03	1.42 ± 0.03	
	*r*_1_	0.9956	0.9963	0.9957	0.9960	0.9961	
**0.16–0.33**	β	7.58 ± 0.28	7.29 ± 0.28	7.16 ± 0.21	7.08 ± 0.20	6.88 ± 0.16	
	*r*_1_	0.9886	0.9884	0.9915	0.99354	0.9954	

**Table 2:** The slope $\alpha =-E_{A}$ and correlation coefficient *r*_2_ for the linear fitting line between ln(*D*_Li_) and $1/\left(k_{B}T\right)$, for different Li^+^ concentration *C*_Li_ (ML).

Concentration *C*_Li_ (ML)	Slope $\alpha =-E_{A}$ (eV)	Correlation Coefficient *r*_2_
0.031	−0.2295 ± 0.0003	0.9999
0.092	−0.2332 ± 0.0004	0.9999
0.153	−0.261 ± 0.002	0.9995
0.235	−0.432 ± 0.002	0.9998
0.281	−0.457 ± 0.001	0.9999
0.332	−0.473 ± 0.001	0.9999
